# Bridging the gap: how education transforms health outcomes and influences health inequality in rural China

**DOI:** 10.3389/fpubh.2024.1437630

**Published:** 2024-10-30

**Authors:** Jingyuan Chen, Longbao Wei, Faiza Manzoor

**Affiliations:** ^1^School of Management, Zhejiang University, Hangzhou, China; ^2^Department of Agricultural Economics and Management, School of Public Affairs, Zhejiang University, Hangzhou, China

**Keywords:** rural residents’ health, health inequality, causel effect, rural China, education

## Abstract

This study focuses on the impact of education on health and health inequalities in rural areas of China. Education significantly enhances economic status and health, driving economic growth and improving public health standards. Integral to the “Healthy China Strategy,” it provides essential guidance for public policy and underscores the need for strategic human capital investments to achieve these goals. The study utilizes data from the China Family Panel Studies (CFPS) spanning 2010–2020 and employs the average educational level within counties as an instrumental variable. The causal impact of education on health and health inequalities is estimated using the two-stage least squares (2SLS) method. The findings reveal a significant positive correlation between enhancing education levels and health improvements. Specifically, after controlling for endogeneity, the duration of individual education significantly improves both subjective and objective health outcomes. It reduces health inequalities, with these effects being more pronounced among women and low-income groups. Mechanistically, education positively impacts health primarily by altering health behaviors and social network levels and reducing health inequalities through socio-economic factors. This paper provides important implications for public policy, suggesting that enhancing educational investments can drive economic development and improve population health standards.

## 1 Introduction

Education is widely acknowledged as a pivotal means to enhance individual economic outcomes and social standing. Amidst ongoing economic growth and social evolution, there is a growing interest in the non-economic benefits of educational investments, particularly regarding their impact on health (1, 2). Numerous recent studies underline a robust association between education and health outcomes, suggesting that higher levels of education substantially improve health metrics. This association has far-reaching implications for public policy, demonstrating that higher educational investment can generate economic and health advantages across communities. Both education and health are vital to socio-economic advancement, where the enhancement of health through education not only provides dual benefits—spurring economic growth and improving public health—but also boosts strategic adjustments in human capital investments (3).

With the implementation of the “Healthy China Strategy,” which aims to improve sustainable social and economic growth by increasing health standards, education plays an important role in accomplishing these goals (4). Additionally, demographic shifts, particularly the rising challenge of an aging population, increase the strain on healthcare systems and impose significant pressures on economic and social welfare systems. Establishing a causal link between education and positive health outcomes could offer strategic insights to counteract these demographic challenges and broadly elevate population health and socio-economic development. Due to disparities in healthcare facilities between rural and urban areas in China, education is critical. Rural residents rely heavily on their educational attainments to access health information, adopt healthier lifestyles, and improve life quality (5). This scenario underscores the critical role of education in health improvement in these underserved areas.

Ross and Mirowsky (6) introduced two theories that explain the variability of education’s effects on health across different demographic groups, the Resource Substitution Theory and second is the Reinforcement of Advantage Theory. The former posits that in socio-economically disadvantaged groups, education is crucial, serving as a compensatory resource that offsets deficiencies in economic and social capital, and health knowledge. This theory suggests that in resource-scarce environments, the value of education is highlighted, thereby having a pronounced positive impact on health among disadvantaged groups. Conversely, the Reinforcement of Advantage Theory suggests that in socio-economically advantaged groups, education compounds existing resources, enhancing health outcomes through synergistic effects that broaden social networks and access to superior healthcare.

**Figure 1:**
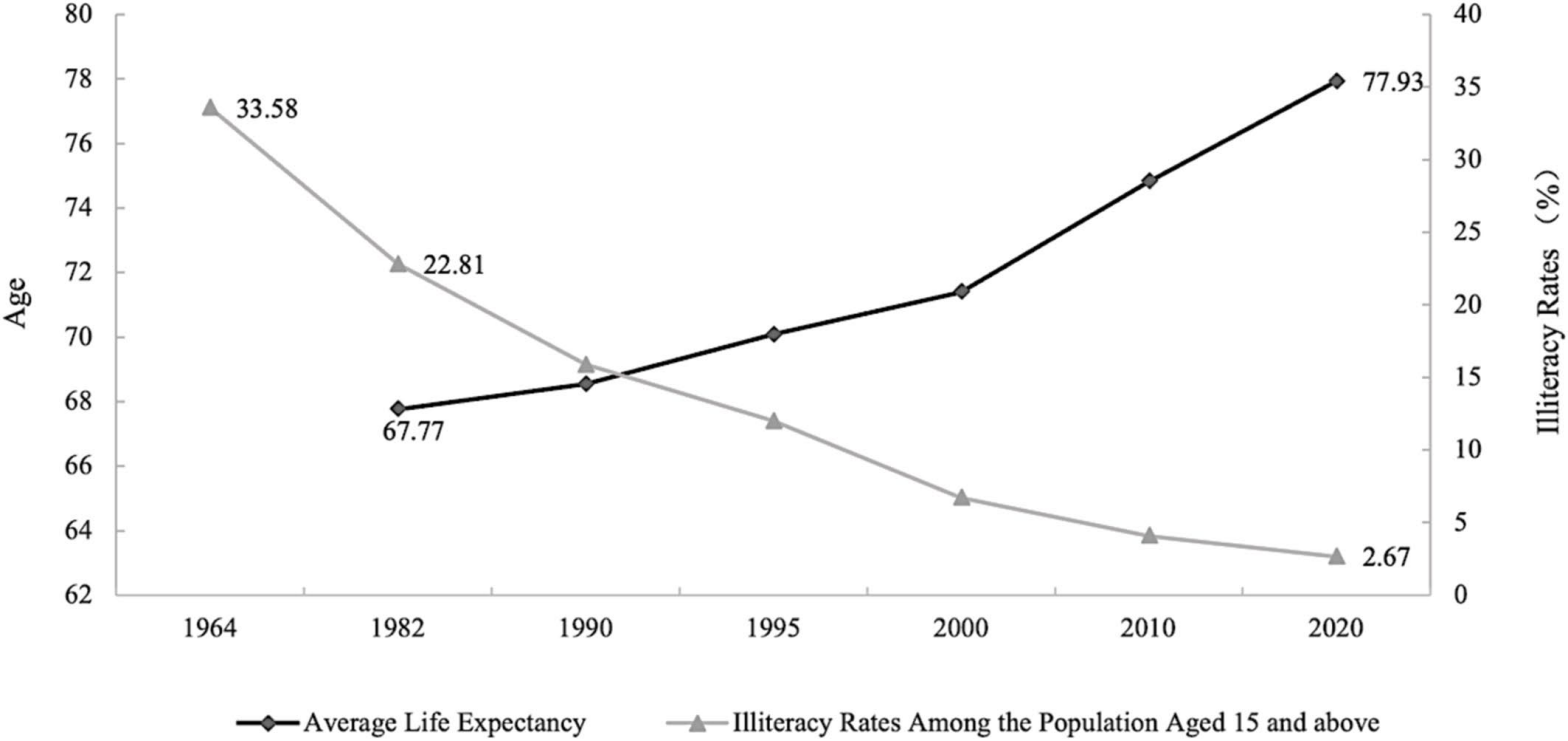
Average life expectancy and illiteracy rates among the population aged 15 and above in China, between 1964–2020. Chinese population census data from 1964 to 2020 (10, 11).

Extensive research supports a positive correlation between education and health (2, 7–9). Figure 1 shows the trend in the average life expectancy of the Chinese population from 1964 to 2020 the illiteracy rate was for those aged 15 and above. From this figure, one might infer a possible negative correlation between the two variables.

However, a reduction in illiteracy or an improvement in educational levels may raise average life expectancy. The positive interaction between educational level and life expectancy could be due to either an increase in life expectancy causing a decrease in illiteracy rates or both being influenced by the same temporal trends or economic levels. To fully realize the positive impact of education on health, it is crucial to establish the causal relationship between education and health (2). However, establishing a causal relationship between these variables remains challenging due to potential third-party influences, reverse causality, and unobservable heterogeneities that may confound this relationship. Addressing these methodological hurdles, recent studies have leveraged quasi-experimental designs and instrumental variables such as local average education levels and compulsory education laws to isolate the causal impact of education on health (12–14).

Despite these developments, consensus on the impact of education on health in China remains elusive, with research yielding conflicting results. However, studies focusing on China have not yet reached a consensus. Some research suggests that education significantly improves individual health levels (15–17), primarily through interventions in health behaviors (18). Other studies show that education has little to no effect on health, or that no causal relationship exists between the two (7). Similarly, neither subjective reports of physical health, mental well-being, or health problems, nor objective physical measures like obesity, overweight, or underweight are influenced by education, with results remaining robust across different sample distinctions by gender, urban–rural status, and changes in regression equation orders and bandwidths (19). There is also research suggesting heterogeneity in the health returns of education by gender (20), health indicators (21), and urban–rural characteristics (22), indicating that the impact of education on health may vary from person to person, so current research has not explored deeper into this issue. In addition, there is inadequate focus in existing research on how education influences health inequality at the individual level. Therefore, our study fills this gap and investigates the impact of education on health outcomes and health inequality. The findings not only deepen our understanding of the role of education in health outcomes in rural China but also shed light on the broader implications for educational policies and interventions aimed at reducing health Inequality.

## 2 Methods of the study

### 2.1 Data and sample

The China Family Panel Studies (CFPS), conducted by Peking University’s Institute of Social Science Survey, constitute a comprehensive study of capturing multi-dimensional changes in China’s economy, society, demographics, education, and health across different time points. Employing Computer-Assisted Personal Interviewing (CAPI) techniques ensured the efficiency and quality of data collection. Given China’s diverse regions, CFPS sampling utilized implicit stratification, multistage, multilevel, and population-proportionate probability sampling methods (PPS). Covering 25 provinces, representing 95% of the mainland population, the CFPS sample excludes Hong Kong, Macau, and Taiwan.

This study focuses on adult samples aged 25–85 residing in rural areas. Individuals under 25 are excluded, as most individuals’ education levels stabilize by age 25. The sample includes individuals aged 25 or older at the 2010 baseline survey, born no later than 1985, excluding those still in education during subsequent survey rounds. Samples above 85 are excluded due to physiological differences (21). Rural classification is based on permanent residence, aligning with the study’s focus on health inequalities stemming from educational and health resource disparities across regions. A binary dummy variable accounts for urban–rural migrants to control for migration effects. Data cleaning involves excluding observations with missing key variables and imputing missing values for non-key variables. Provinces and occupational samples with insufficient representativeness are removed. The final dataset comprises 58,169 rural adult samples, with 28,407 male and 29,762 female samples.

### 2.2 Description of the variables

Each wave of the CFPS collects data related to health, which can be categorized into subjective and objective health variables. Subjective health variables include (1) self-rated health (“How would you rate your health?”); (2) health compared to the previous year (“How do you feel about your health compared to last year?”); (3) health relative to peers (“How do you feel about your health compared to people of your age?”); (4) scores on the psychological depression scale. Objective health variables include (1) Body Mass Index (BMI); (2) the presence of chronic diseases within the past year; and (3) physical mobility impairments (Activities of Daily Living—ADL). In existing studies, scholars have utilized various health indicators to measure health status, such as self-rated health (23), physical and cognitive function levels (24) BMI, cognitive function, and medical expenditures (25). Given that different health metrics only reflect certain aspects of health the discrepancy between subjective and objective health metrics can change based on the socioeconomic factors of the individual (26). Therefore, this study employs both subjective and objective indicators to assess health. Subjectively, the study considers individuals’ self-rated health and mental health, while objectively, it examines whether BMI is within the standard range and whether an individual has chronic diseases. It is noted that ADL, often used in studies, is not employed as an objective health indicator here due to its specific application in CFPS to individuals aged 65 and over, while the average age of the study’s subjects is around 49. Including this demographic could introduce sample selection bias. Detailed below (1) and (2) are the outcome variables of this study.

#### 2.2.1 Levels of health

##### 2.2.1.1 Self-rated health

Self-rated health is an accessible and authoritative health indicator, known to predict mortality and morbidity effectively (27) and widely used in epidemiological, sociological, and health economics research (28). In the CFPS database, it is obtained through the question, “How do you feel about your health level?” Notably, the response options have varied over the years, with a change in 2012 that does not impact cross-sectional comparisons significantly but does affect longitudinal analyses. There is no direct evidence suggesting that these changes in response options significantly affect longitudinal comparisons, and existing studies have not made special adjustments. This study takes the same approach and, for clarity, inversely codes this variable, with larger numbers indicating better self-rated health.

##### 2.2.1.2 Mental health

In recent years, more researchers have focused on the impact of several psychological factors, including non-cognitive skills on individuals. The CFPS psychological scale primarily measures respondents’ personality traits, parent–child relationships, and subjective attitudes. This study uses the level of depression to reflect mental health, employing two scales in CFPS, i.e., the Kessler Psychological Distress Scale (K6) and the Center for Epidemiologic Studies Depression Scale (CES-D). The K6 scale was used in 2010 and 2014, while the CES-D scale was used in other years. Due to the non-comparability of scores between these scales, this study binary transforms the results. Specifically, the K6 scale categorizes depression into low risk (0–12 points) and high risk (13–24 points), while the CES-D categorizes it into four levels. For this study 12 and 20 points are thresholds for the K6 and CES-D scales, respectively. Scores below these thresholds indicate good mental health (mehealth = 1), and scores above indicate poor mental health (mehealth = 0). Given the loss of information inherent in binary transformations, this study also tests the robustness of its findings using continuous scores from the K6 and CES-D scales as proxies for mental health.

##### 2.2.1.3 BMI index

The Body Mass Index is calculated as weight in kilograms divided by the square of height in meters. This metric assesses whether an individual’s weight is within a healthy range. Due to frequent missing data on height in the CFPS, this study uses the median height from different years for everyone. Weight data, less frequently missing, are linearly imputed and trimmed at 1% to remove outliers. BMI does not have a monotonous continuous health implication; therefore, this study creates a binary variable representing health levels. According to the health standards published by the National Health and Family Planning Commission of the People’s Republic of China in 2013, BMI is categorized into obese (BMI >28), overweight (24 < BMI ≤ 28), normal weight (18.5 ≤ BMI < =24), and underweight (BMI < 18.5). This study constructs a binary dummy variable (bmihealth), where 1 indicates a BMI within the normal range, and 0 specifies otherwise (obese, overweight, and underweight).

##### 2.2.1.4 Presence of chronic diseases

This binary variable is set based on responses to whether an individual has been diagnosed with a chronic disease in the last six months. A value of 1 indicates the presence of at least one chronic disease, and 0 specifies no chronic diseases.

#### 2.2.2 Levels of health inequality

Most current research indicators for inequality use group-level inequality metrics, such as the Gini coefficient, Erreygers index, Atkinson index, etc. To delve deeper into the micro-level factors of inequality, this study chooses individual-level inequality indices. Yitzhaki (29) introduced the concept of the relative deprivation curve to characterize and analyze the distribution of income and wealth (30). This is a type of inequality measure based on individual-level comparisons, similar to the Kakwani Index (31) and the Podder Index (30). Yitzhaki provided an interpretation of the Gini coefficient through the lens of (32) theory of relative deprivation. Kakwani’s approach parallels that of (33), in which everyone can compare themselves with others randomly drawn from the overall population. A key advantage of this method over the Gini coefficient is its ability to effectively address the Gini’s insensitivity to the lower tail of the income distribution. These indices have been widely applied in the study of income and consumption inequality (34, 35). Among these, the Yitzhaki index’s advantage is that its weighted average is the absolute Gini coefficient, however, it is sensitive to sample size and dependent variable distribution and does not satisfy dimensionlessness and normalization properties. The Podder index overcomes the sensitivity of the dependent variable distribution but does not address normalization issues. The Kakwani index satisfies normalization and dimensionlessness properties, and another advantage is that the regional average is equivalent to the Gini coefficient, so it is sensitive to fluctuations in lower-level groups (34). This study considers the Kakwani index for its excellent characteristics of dimensionlessness and normalization, constructing a health-relative deprivation index based on the Kakwani index in the main regression to measure health inequality levels. Due to the data distribution sensitivity of the Kakwani index, this study further uses the Yitzhaki and Podder indices for robustness testing to complement the strengths of the indices. Moreover, this study utilizes the corresponding relationship between the Kakwani index and the Gini coefficient, calculating the weighted average of the Kakwani index at different county levels to obtain county-level health Gini coefficients, thus enabling comparative analysis of health inequality levels at the regional level, closely linking micro-level individual relative deprivation with macro-level inequality. The specific calculation formulas for the three indices are as follows:

Define a group H containing N individuals, with the health vector of the group arranged in ascending order of health levels, i.e., $h\leq h_{2}\leq \cdots \leq h_{i}\leq \cdots \leq h_{N}$ . The relative deprivation index for the health of an individual i is defined a

(1) $$\begin{array}{l} \mathrm{Health}\_Kak\_RD\left(h,h_{k}\right)=\\ \frac{1}{N\mu_{h}}\overset{N}{\underset{i=k+1}{\sum }}\left(h_{i}-h_{k}\right)=\theta_{h_{k}}^{+}\left[\left(\mu_{h_{k}}^{+}-h_{k}\right)/\mu_{h}\right]\; \end{array}$$

(2) $$\mathrm{Health}\_Yit\_RD\left(h,h_{k}\right)=\frac{1}{N}\overset{N}{\underset{i=k+1}{\sum }}\left(h_{i}-h_{k}\right)=\theta_{h_{k}}^{+}\left[\left(\mu_{h_{k}}^{+}-h_{k}\right)\right]$$

(3) $$\begin{array}{l} \mathrm{Health}\_\mathrm{Pod}\_RD\left(h,h_{k}\right)=\\ \frac{1}{N}\overset{N}{\underset{i=k+1}{\sum }}\left(\ln h_{i}-\ln h_{k}\right)=\theta_{h_{k}}^{+}\left[\left(\mu_{\ln h_{k}}^{+}-\ln h_{k}\right)\right]\; \end{array}$$

In Equations 1–3, $\mu_{h}$ is the overall mean health level, $\mu_{h_{k}}^{+}$ is the mean health level of individuals in group H whose health levels are higher than $h_{k}$ , and $\theta_{h_{k}}^{+}$ is the proportion of individuals in group H whose health levels are higher than $h_{k}$ . Formulas 1–3 correspond to the individual health relative deprivation indices calculated based on the Kakwani index, Yitzhaki index, and Podder index, respectively, indicating the level of individual health inequality.

The core explanatory variable in the main regression of this study is the duration of education (eduyear). In robustness testing, the study further differentiates by educational stage. The first approach estimates the overall health returns of each additional year of education, while the second approach distinguishes the heterogeneous effects of different educational stages on individual health levels. In the original CFPS database, individual education levels are represented by an ordinal categorical variable ranging from 1 to 9, where 1 represents illiteracy/semi-literacy; 2 represents completion of elementary education; 3, junior high school; 4, high school; 5, vocational high school/general high school/technical school; 6, junior college; 7, bachelor’s degree; 8, master’s degree; 9, doctoral degree. For people having conflicting records throughout time, the lower record is regarded as authoritative. Given that samples with a bachelor’s degree or higher are low (less than 1%), for balance considerations, samples with junior college, bachelor’s, master’s, and doctoral degrees are combined into one category, labeled “junior college and above.”

Referencing existing literature and taking into account the potential factors influencing individual health and the availability of data, the control variables used in this study are categorized into four main areas: (1) demographic characteristics, including individual age (*age*), age squared (*age^2^*), gender (*gender*), and marital status (*marriage*); (2) household economic characteristics, such as occupational features (*jobtype*), political affiliation (*party*), household size (*famsize*), the logarithm of *per capita* annual household expenditure (*lnexpense*), and whether the individual migrates to an urban area in the next observation period (*immigrant*); (3) living and sanitation conditions, including the availability of tap water (*ckwater*), whether the toilet is a flush toilet (*toilet*), and whether garbage is centrally disposed of (*cgdisposal*); and (4) medical conditions, such as whether the individual has health insurance (*insurance*). Additionally, to account for regional heterogeneity and time trend effects, the study also controls for province and year fixed effects.

### 2.3 Descriptive statistics

Table 1 presents the descriptive statistics for the main variables used in this study. First, the statistical characteristics of variables related to health levels and health inequality are focused upon. These variables include self-rated health (SRH), mental health (mehealth), CES-D scores, K6 scores, whether BMI is within the normal range (bmihealth), whether chronic diseases are present (disease), and three health inequality indices (RD_kak, RD_yit, and RD_pod). The mean of self-rated health (SRH) across all samples is 3.094, indicating that individuals generally rate their health above average. A relatively low variance (1.334) suggests modest fluctuations in self-rated health among individuals. The mean of mental health (mehealth) is 0.82, indicating that nearly 30% of rural residents experience mental health issues. The mean scores for the CES-D and K6 scales are 13.638 and 9.295, respectively, showing a wide range of variation and significant differences in these dimensions of psychological health among individuals. The mean of BMI health status (bmihealth) is 0.625, indicating that most individuals’ BMI is within the normal range. The presence of chronic diseases (disease) as a binary variable has a mean of 0.156, indicating that 15.6% of individuals in the sample have had at least one chronic disease in the past six months. From the perspective of health inequality indices, the average values of the three indices (RD_kak, RD_Yit, RD_Pod) are 0.185, 12.829, and 0.248, respectively. RD_Yit shows high variance, indicating sensitivity to data fluctuations, while RD_kak is more stable compared to the other two indices.

**Table 1:** Descriptive statistics of key variables.

Variable	Description	Variable type	Mean	Standard deviation	Minimum	Maximum
SRH	Self-rated health, where 5 = very healthy.	Ordinal categorical variable	3.094	1.334	1	5
mehealth	Mental health, where 1 = healthy.	Binary	0.820	0.384	0	1
CESD	Scores from the Center for Epidemiologic Studies Depression Scale.	Continuous	13.638	7.857	0	38
K6	Scores from the Kessler Psychological Distress Scale.	Continuous	9.295	3.794	6	24
bmihealth	Whether BMI is within the healthy range (18.5–24).	Binary	0.625	0.484	0	1
cdhealth	Whether there has been a chronic disease diagnosed in the last six months, yes = 1.	Binary	0.156	0.591	0	1
RD_kak	Health relative deprivation level based on the Kakwani index.	Continuous	0.185	0.211	0	1
RD_Yit	Health relative deprivation level based on the Yitzhaki index.	Continuous	12.829	14.658	0	69.474
RD_Pod	Health relative deprivation level based on the Podder index.	Continuous	0.248	0.517	0	4.152
Eduyear	Individual’s years of education.	Continuous	5.780	4.265	0	19
Edutype1	Illiterate/semi-literate.	Binary	38.0%	0.485	0	1
Edutype2	Elementary.	Binary	26.3%	0.440	0	1
Edutype3	Junior high school.	Binary	26.2%	0.440	0	1
Edutype4	Senior high school.	Binary	7.9%	0.269	0	1
Edutype5	Junior college and above.	Binary	1.6%	0.126	0	1
Gender	Gender, male = 1.	Binary	0.500	0.500	0	1
Age	Age (centered).	Continuous	0.011	9.814	−22.182	20.818
Age^2^	Squared age (centered).	Continuous	96.304	105.952	0.331	492.036
Marriage	Whether married, yes = 1.	Binary	0.928	0.258	0	1
Party	Whether a member of the Communist Party, yes = 1.	Binary	0.063	0.243	0	1
Famsize	Family size.	Continuous	4.599	1.958	1	21
Immigrant	Whether migrated to urban areas subsequently, yes = 1.	Binary	0.110	0.313	0	1
Jobtype1	Unemployed	Binary	15.2%	0.359	0	1
Jobtype2	Enterprise or institution leader.	Binary	2.1%	0.145	0	1
Jobtype3	Professional and technical staff.	Binary	1.8%	0.133	0	1
Jobtype4	Clerk or sales staff.	Binary	7.1%	0.173	0	1
Jobtype6	Agricultural producer.	Binary	58.7%	0.492	0	1
Jobtype7	Manufacturing producer.	Binary	15.0%	0.210	0	1
lnexpense	Logarithm of *per capita* total family expenditure.	Continuous	10.382	0.877	2.303	15.450
Ckwater	Whether the water used for cooking is tap water (or purified water), yes = 1.	Binary	0.549	0.498	0	1
Toilet	Whether the toilet is a flush toilet, yes = 1.	Binary	0.428	0.428	0	1
Cgdisposal	Whether there is a centralized garbage disposal site, yes = 1.	Binary	0.467	0.467	0	1
Insurance	Whether the individual has health insurance, yes = 1.	Binary	0.903	0.297	0	1
*N*	Sample size		58,169			

Regarding education levels, rural residents have an average of 5.780 years of education, roughly equivalent to elementary education. Breaking it down by educational stages, 38.0% of the sample has not received elementary education, 26.3% have completed elementary education, 26.2% have completed compulsory education, and less than 10% have received high school or higher education. This shows that elementary education remains the most common level of education among the rural sample at this stage. As to control variables, the average age of the sample is 47.3 years, and this study standardizes all samples to this age for analytical consistency. Regarding gender, the sample has an equal ratio of males (0.500) to females (0.500), indicating no significant bias in gender composition. In terms of occupation, about 58.7% of the individuals are engaged in agricultural work, while less than 4% are enterprise leaders or professionals. Concerning marital status 92.8% of individuals are married, revealing that marriage is a common phenomenon in rural areas. Politically, 6.3% of the people are members of the Communist Party. In terms of health behaviors, the smoking rate in the entire sample is 32.1%, and the drinking behavior rate is 13.6%, which is relatively low. As to exercise levels, the average weekly exercise duration is 2.061 h. These statistics provide preliminary insights into the health and educational socio-economic conditions of rural residents in China.

### 2.4 Tools of estimation

#### 2.4.1 Baseline regression

This paper initially conducts a baseline regression to examine the empirical relationship between educational human capital and health and its inequalities. The baseline regression models are presented in Equations 4, 5.

(4) $$\mathrm{Healt}h_{\mathrm{it}}=\alpha_{0}+\alpha_{1}\mathrm{eduyea}r_{i}+\alpha_{j}f\left(\mathrm{ag}e_{\mathrm{it}}\right)+\delta_{j}X_{\mathrm{it}}+\mu_{\mathrm{it}}$$

(5) $$RD_{\mathrm{it}}=\beta_{0}+\beta_{1}\mathrm{eduyea}r_{i}+\beta_{j}f\left(\mathrm{ag}e_{\mathrm{it}}\right)+\gamma_{j}X_{\mathrm{it}}+\varepsilon_{\mathrm{it}}$$

In Equation 4, $\mathrm{Healt}h_{\mathrm{it}}$ represents the individual health level, which is denoted using four proxy variables such as self-rated health (*SRH*), mental health (*mehealth*), whether BMI is within a healthy range (*bmihealth*), and whether the individual has chronic diseases (*cdhealth*). SRH is an ordinal categorical variable, it is estimated by using an Ordered Probit Model. Meanwhile, *mehealth*, *bmihealth*, and *cdhealth*, being binary dummy variables, are estimated using the Probit Model.

In Equation 5, $RD_{\mathrm{it}}$ represents the level of health inequality, specifically the individual health relative deprivation index. $\mathrm{eduyea}r_{i}$ denotes the individual’s years of education, serving as the core explanatory variable. The function *f* (∙) represents a multilevel linear function of individual age age, with the highest order set to 2, in line with existing health economics literature (20), to control for the impact of age on health. *X* represents covariates, including gender (*gender*), political affiliation (*party*), marital status (marriage), family size (*famsize*), and subsequent migration (*immigrant*). Furthermore, to account for potential time trends and regional heterogeneity regional and Year-fixed effects are controlled. α⋅ ， β⋅ ， $\delta_{j}，\gamma_{j}$ are parameters to be estimated, while $\mu_{\mathrm{it}}$ and $\varepsilon_{\mathrm{it}}$ are error terms.

#### 2.4.2 Causal inference: IV two-stage regression design

Establishing a causal relationship between education and health presents significant endogeneity issues, primarily for three reasons. First, the intervention of third-party factors complicates the identification of causality. Variables such as family background, individual ability, and time preferences can influence both an individual’s level of education and their health outcomes. For instance, an individual from a more affluent family might receive a higher level of education and, due to the advantages of family resources, may also enjoy better health conditions. In this case, the observed relationship between education and health could be partially or entirely driven by these third-party factors, rather than a direct effect of education on health. Second, the presence of reverse causality adds further complexity to the analysis. Childhood illnesses, for example, can impact the level of education an individual attains later in life, as health issues may lead to missed school, learning difficulties, or even early dropout. In such situations, health becomes a determinant of education, rather than vice versa. This indicates that when assessing the potential impact of education on health, it is crucial to account for the reverse effect of health on education. Finally, both education and health may be influenced by unobservable heterogeneity. This unobserved heterogeneity could include lifestyle choices, genetic predispositions, or other unmeasured variables that could simultaneously affect education and health outcomes. The challenge here is that, even if a strong correlation between education and health is observed, determining the true causal relationship becomes difficult if these unmeasured factors cannot be controlled for or accounted for.

From a methodological perspective, the endogeneity issue means that direct estimation using OLS would inevitably be biased (36). For this reason, some studies have considered using twin data to control unobservable factors (37), compulsory education laws (13, 38), minimum working age regulations (39) the educational level of spouses (40), and the educational level of mothers (41) as instrumental variables for education, employing two-stage least squares regression to estimate the causal effects of education on health. In recent years, quasi-experimental methods have been gaining attention and application in exploring causal effects in the field of education’s health returns, with studies Zhao et al. (42) using Difference-in-Differences—Two-Stage Least Squares estimation methods, Guo et al. (3) and Li et al. (21) using the Regression Discontinuity (RD) framework to estimate the impact of education on health. In terms of data selection, these studies often utilize nationally representative micro databases, such as the Chinese General Social Survey (CGSS) data used by Chen et al. (43) the CFPS 2010—2018 data used Guo et al. (44), and the China Health and Retirement Longitudinal Study (CHARLS) as well as the 2005 1% population sample data by Liu and Xu (45).

Given these challenges, this study adopts a two-stage least squares (2SLS) regression approach with instrumental variables to establish the causal relationship between education and health, thereby reducing bias and controlling for confounding factors. Based on the basic selection criteria for instrumental variables, this study uses the county-level average years of schooling, excluding the individual, as an instrumental variable for the individual’s years of schooling. This is because the average level of education in a county reflects the region’s economic and social development, as well as its emphasis on educational culture, which could lead to individuals with higher education levels, nevertheless, the regional average education level does not directly affect an individual’s health outcomes. Likewise, an individual’s health level does not impact the regional average education level. Considering the impact of sample size, as CFPS surveys typically select only a few households per village, using the village as a unit would result in fewer and unstable samples, thus the county is chosen as a unit to enhance the stability of the instrumental variable.

The study employs a two-stage regression method for estimation, where the first-stage regression estimates the impact of the county’s average education level, excluding the individual, on individual years of education, as shown in Equation 6:

(6) $$\mathrm{eduyea}r_{\mathrm{it}}=\lambda_{0}+\lambda_{1}\mathrm{edumea}n_{\mathrm{it}}+\lambda_{i}f\left(\mathrm{ag}e_{\mathrm{it}}\right)+\pi_{i}X_{\mathrm{it}}+\nu_{\mathrm{it}}$$

Here, $\mathrm{eduyea}r_{\mathrm{it}}$ is the individual’s years of education, $\mathrm{edumea}n_{\mathrm{it}}$ is the instrumental variable, i.e., the average years of education of other individuals in the county, $\lambda_{0}-\lambda_{i}$ are parameters to be estimated, and $\nu_{\mathrm{it}}$ is the error term. The other settings remain consistent with Equations 4–5. This two-stage estimation method tests the impact of education on health, specifically, the first stage uses Equation 6 to estimate $\mathrm{eduyea}r_{\mathrm{it}}$ to obtain ${\hat{\mathrm{eduyear}}}_{\mathrm{it}}$ , which is then entered into Equations 4–5 to get the second-stage estimates for $\alpha_{1}$ and $\beta_{1}$ .

## 3 Analysis and results

Table 2 reports the baseline regression results of educational human capital on the immediate utility of subjective health. The results include six models, where models (1–3) utilize self-rated health as the dependent variable in an Ordered Probit Model regression, and models (4–6) use individual mental health as the dependent variable in a Probit Model regression. In models (1–3) self-rated health as an ordinal categorical dependent variable, is used with higher values indicating better individual health conditions. As a continuous explanatory variable, the duration of education is used to predict the probabilities of different health grades. The first-row coefficients for different self-rated health levels represent the Average Marginal Effects (AME). Model (1) does not include control variables, whereas models (2) and (3) incorporate a set of control variables and fixed effects for regions and time, respectively. It is found that with all predetermined variables and fixed effects controlled, an additional year of education decreases the probability of being “unhealthy” (*SRH* = 1) by an average of 0.4%, indicating a significant positive effect of increased educational duration on improving the health of individuals with the poorest health status. For individuals with health levels “average” or “fairly healthy” (*SRH* = 2, 3), with all variables controlled, an additional unit increase in education duration decreases these probabilities by an average of 0.2 and 0.1%, respectively.

**Table 2:** Baseline regression results on the utility of education on subjective health status.

	SRH			Mental health		
	Ordered probit(dy/dx)			Probit(dy/dx)		
	(1)	(2)	(3)	(4)	(5)	(6)
Eduyear				0.013*** (0.000)	0.010*** (0.001)	0.009*** (0.001)
SRH = 1	−0.008*** (0.000)	−0.005*** (0.000)	−0.004*** (0.000)			
SRH = 2	−0.004*** (0.000)	−0.002*** (0.000)	−0.002*** (0.000)			
SRH = 3	−0.001*** (0.000)	−0.001*** (0.000)	−0.001*** (0.000)			
SRH = 4	0.004*** (0.000)	0.002*** (0.000)	0.002*** (0.000)			
SRH = 5	0.010*** (0.000)	0.006*** (0.000)	0.004*** (0.000)			
Age		−0.044*** (0.001)	−0.024*** (0.001)		−0.002*** (0.000)	−0.002*** (0.000)
Age^2^		0.000 (0.000)	0.000** (0.000)		0.000** (0.000)	0.000** (0.000)
Control variables	No	Yes	Yes	No	Yes	Yes
Province fixed effects	No	No	Yes	No	No	Yes
Year fixed effects	No	No	Yes	No	No	Yes
$\chi^{2}$	577.6	4369.6	11285.2	690.2	996.4	1,449
Prob> $\chi^{2}$	0.000	0.000	0.000	0.000	0.000	0.000
N	58,169					

Standard errors are in parentheses; * indicates significance levels * *p* < 0.1, ** *p* < 0.05, *** *p* < 0.01.

Additionally, for higher health levels (“healthy” and “very healthy”) (*SRH* = 4, 5), each additional year of education increases the probability of being healthy (*SRH* = 4) by 0.2% and very healthy (*SRH* = 5) by 0.4% after controlling for all variables. These conclusions all suggest that increasing educational years significantly enhances self-rated health evaluations. Comparing models (1), (2), and (3), the marginal utility of education duration on self-rated health decreases as more control variables are introduced, indicating that factors other than education also influence health. However, the relationship between education duration and self-rated health remains significant and directionally consistent. These results strengthen the evidence of the relationship between improved educational levels and enhanced self-rated health status. Statistical significance (marked by asterisks in the coefficients) and the robustness of the marginal effects, even after controlling for other variables, point to education as a key factor in improving individual health probabilities.

Models (4–6) display results using the Probit Model to estimate the immediate utility of education duration on individual mental health levels. Here, the first-row coefficients represent the Average Marginal Effects (AME). Model (4) does not include control variables, while models (5) and (6) respectively incorporate a set of control variables and fixed effects for regions and time. In model (4) the average marginal effect is 0.013, meaning that for each additional year of education, the probability of rural residents being mentally healthy significantly increases by 1.3%. When considering other control variables and regional and Year-fixed effects, this drops to 0.9%, but remains significant at the 0.01 level, indicating a robust positive impact of educational human capital on maintaining mental health, consistently observed across multiple model setups.

Furthermore, in models (3) and (6), the coefficients for both the first and second powers of age are significant, implying a significant “U”-shaped effect of age on individual self-rated health and mental health, with the turning point for self-rated health at age 79.5 and for mental health at age 61.5. This indicates that with other variables controlled the level of mental health increases with age between 61.5 and 79.5 years, while self-rated health decreases with increasing age until 79.5 years, after which both increase with age.

Table 3 depicts the regression results of educational human capital on the immediate utility of individuals’ objective health. Initially, models (1–3) use whether an individual’s BMI index is within the standard range as the outcome variable, where 1 represents being within the standard range. From the regression results, the Average Marginal Effect (AME) of years of education is consistently positive, indicating that as years of education increase, the probability of an individual’s BMI index being within the healthy range also increases. Model (2) shows that, after controlling for variables other than province-fixed effects and Year-fixed effects, each additional year of education increases the probability of having a BMI within the standard range by an average of 0.7%. This result suggests that education plays a positive role in maintaining a healthy weight, likely because higher levels of education are often accompanied by better health knowledge and healthier lifestyle habits. However, Model (3) indicates that although this effect remains positive when controlling for time and province-fixed effect, it is no longer significant. This might imply that the positive impact of education on BMI could partially be due to changes over time or may relate to regional differences in dietary habits, lifestyles, and cultural factors.

**Table 3:** Baseline regression results on the utility of education on objective health status.

	Bmihealth			Chronic disease		
	Probit(dy/dx)			Probit(dy/dx)		
	(1)	(2)	(3)	(4)	(5)	(6)
eduyear	0.006*** (0.000)	0.007*** (0.001)	0.001 (0.001)	−0.006*** (0.000)	−0.004*** (0.001)	−0.003*** (0.001)
Age		−0.004*** (0.000)	−0.001*** (0.000)		0.006*** (0.000)	0.006*** (0.000)
Age^2^		0.000 (0.000)	0.000*** (0.000)		0.000 (0.000)	0.000** (0.000)
Control variables	No	Yes	Yes	No	Yes	Yes
Province fixed effects	No	No	Yes	No	No	Yes
Year fixed effects	No	No	Yes	No	No	Yes
$\chi^{2}$	160	248.9	2423.9	141.9	1355.6	1608.3
Prob > $\chi^{2}$	0.000	0.000	0.000	0.000	0.000	0.000
*N*	58,169					

Standard errors are in parentheses; * indicates significance levels * *p* < 0.1, ** *p* < 0.05, *** *p* < 0.01.

Furthermore, models (4–6) use whether an individual has a chronic disease as the dependent variable, where 1 indicates the presence of a chronic disease. According to these models, the AME of years of education is negative, indicating that the risk of acquiring a chronic illness decreases as years of education increase. The coefficient in Model (6) is −0.003, demonstrating that each additional year of education drops the probability of having a chronic disease by an average of 0.3%. This finding further underscores the importance of education in promoting objective health.

Table 4 shows the immediate effects of years of education on the level of health inequality among residents. The level of relative health deprivation measured using the Kakawin index, serves as an indicator of individual health inequality. Three models are presented here such as Model (1), which controls no other variables or regional and temporal effects, Model (2) controls other variables; and Model (3) handles all variables including fixed effects for time and region.

**Table 4:** Baseline regression results on the utility of education on health inequality.

	Relative health deprivation level (Kakwani index)		
	OLS		
	(1)	(2)	(3)
Eduyear	−0.006*** (0.000)	−0.004*** (0.000)	−0.003*** (0.000)
Gender		−0.030*** (0.004)	−0.031*** (0.004)
Age		0.004*** (0.000)	0.003*** (0.000)
Age^2^		0.000* (0.000)	0.000 (0.000)
Constant	0.216*** (0.002)	0.147*** (0.012)	0.506*** (0.161)
Control variables	No	Yes	Yes
Province fixed effects	No	No	Yes
Year fixed effects	No	No	Yes
$\chi^{2}$	1729.19	6198.25	7454.21
Prob > $\chi^{2}$	0.000	0.000	0.000
N	58,169		

Standard errors are in parentheses; * indicates significance levels * *p* < 0.1, ** *p* < 0.05, *** *p* < 0.01.

From the results of Model (1), the coefficient for years of education is −0.006, significant at the 0.01 level. This indicates that without controlling for any other variables each additional year of education on average reduces the level of relative health deprivation by 0.6%. This preliminary result suggests that education may help improve the relatively disadvantaged position of individual health. After incorporating other control variables, the coefficient for years of education becomes −0.004, still significant at the 0.01 level. This demonstrates that even after controlling for the impact of other variables the role of education in reducing health inequality persists. However, compared to Model (1) the absolute value of the coefficient decreases, which might imply that part of the impact of education on health inequality is indirectly through other variables.

In Model (3), further controlling for regional and temporal fixed effects, the coefficient for years of education decreases to −0.003 and remains significant at the 0.01 level. This indicates that even after controlling for all other factors the direct impact of education on health inequality is still significant, but the magnitude of the effect is reduced. This could be due to some unobserved factors allied to education included in the regional and Year fixed effects, which may be associated with health inequality.

It is observed in Models (1–3) that the coefficient for gender is consistently negative and significant at the 0.01 level, showing that women are more likely than males to be at a health disadvantage, emphasizing gender inequality in health among rural residents. The coefficient for age is positive and significant at the 0.01 level in Models (2) and (3), suggesting that health inequality worsens with age. However, the coefficient for the squared term of age is not significant, indicating that the relationship between age and health inequality may not be quadratic.

Overall, there is a significant negative correlation between years of education and the level of individual relative health deprivation even after controlling for other variables and regional and temporal effects. This relationship persists and shows education’s role in reducing the relative disadvantaged status of individual health.

Table 5 shows the first-stage regression results using the average years of education at the county level as an instrumental variable for individual years of education, under two different model settings. In Model (1) without controlling for other variables, only the impact of the average county education duration on individual education duration was considered. The results indicate a positive correlation between the average county education duration excluding the individual and individual years of education with a coefficient of 0.533 significant at the 1% level (*p* < 0.01). This implies that all else being constant, a one-unit increase in the average county education duration results in an average increase of 0.533 years in individual education duration. Model (2) incorporates control variables including fixed effects for time and region, providing a more refined calibration of the results. In this model, the average county education duration coefficient slightly decreases to 0.497. It is highly significant at the 1% level, indicating that the effectiveness of this instrumental variable remains unchanged even after controlling for other factors. The negative coefficients of age and its squared term indicate an inverse relationship between age and individual years of education, suggesting that the incremental increase in education duration diminishes with age. In both models, the χ^2^ statistic and probability values indicate a good overall fit of the model and statistical significance. The control variables such as Province fixed effects and temporal effects significantly improve the model’s explanatory power, as reflected in the higher χ^2^ statistic and stable coefficient estimates in Model (2) compared to Model (1). These results underscore the significant impact of the county educational environment on individual educational achievements, and this impact remains robust even after controlling for potential confounding factors. The findings emphasize the importance of the social educational environment in shaping individual educational trajectories. They also validate the use of average county education duration as an instrumental variable for individual years of education.

**Table 5:** IV-2SLS regression results of education on health status: first stage.

		Eduyear	
		OLS	
		(1)	(2)
Average eduyear at the county level excluding the individual (IV)		0.533*** (0.010)	0.497*** (0.011)
Constant		2.948*** (0.066)	2.413*** (0.118)
Control variables		No	Yes
Province fixed effects		No	Yes
Year fixed effects		No	Yes
$\chi^{2}$		2799.19	2898.77
Prob > $\chi^{2}$		0.000	0.000
N		58,169	

Standard errors are in parentheses; * indicates significance levels * *p* < 0.1, ** *p* < 0.05, *** *p* < 0.01.

Table 6 presents the second-stage results of the instrumental variable estimation for the impact of years of education on subjective health levels. In Models (1) and (2) the effects of education duration on self-rated health were analyzed using an Ordered Probit model regression. The results of Model (1) show that, after fixing for endogeneity, an increase in years of schooling greatly reduces the likelihood of persons being in poor health and increases the likelihood of being in good health. All results are statistically significant (*p* < 0.01). However, in Model (2) with the inclusion of control variables the impact of years of education on certain health levels becomes nonsignificant. This suggests that other control variables may capture the health effects probably generated by years of education, but the sign and overall trend of the coefficients remain consistent.

**Table 6:** IV-2SLS regression results of education on subjective health status: second stage.

		SRH		Mental health	
		Ordered Probit(dy/dx)		Probit(dy/dx)	
		(1)	(2)	(3)	(4)
Eduyear (fitted values)				0.036*** (0.002)	0.037*** (0.003)
SRH = 1		−0.025*** (0.002)	−0.001* (0.003)		
SRH = 2		−0.011*** (0.001)	0.000 (0.001)		
SRH = 3		−0.003*** (0.000)	0.000 (0.000)		
SRH = 4		0.011*** (0.001)	0.001* (0.001)		
SRH = 5		0.029*** (0.002)	0.002** (0.003)		
Age			−0.026*** (0.001)		−0.003*** (0.000)
Age^2^			0.000** (0.000)		0.000*** (0.000)
Control variables		No	Yes	No	Yes
Province fixed effects		No	Yes	No	Yes
Year fixed effects		No	Yes	No	Yes
$\chi^{2}$		466.7	10125.5	768.2	1652.3
Prob> $\chi^{2}$		0.000	0.000	0.000	0.000
N		58,169			

Standard errors are in parentheses; * indicates significance levels * *p* < 0.1, ** *p* < 0.05, *** *p* < 0.01.

Models (5) and (6) use Probit regression to analyze the impact of years of education on mental health. The coefficients for the fitted values of years of education in Models (5) and (6) are 0.036 and 0.037 respectively, both significant at the 1% level. This indicates that years of education have a significant and positive impact on mental health which means that higher levels of education contribute to improving individual mental health states. After controlling for all variables, each additional year of education increases the probability of better mental health by 3.7%.

Table 7 reveals the second-stage regression results using the instrumental variable method to estimate the impact of education on the objective health levels of rural residents. In models (7) to (10), the fitted values of years of education are employed as an explanatory variable to analyze their impact on BMI and the existence of chronic diseases using the Probit model. In models (7) and (8), the explanatory variable is whether BMI is within the standard range, and in models (9) and (10), the explanatory variable is whether an individual has a chronic disease. Models (7) and (9) do not include control variables, whereas models (8) and (10) control for other variables as well as fixed effects for time and region.

**Table 7:** IV-2SLS regression results of education on objective health status: second stage.

	Bmihealth		Chronic disease	
	Probit(dy/dx)		Probit(dy/dx)	
	(7)	(8)	(9)	(10)
Eduyear (fitted values)	0.049*** (0.002)	0.059*** (0.006)	−0.003 (0.002)	−0.012*** (0.003)
Age		−0.004*** (0.001)		0.006*** (0.000)
Age^2^		0.000*** (0.000)		0.000 (0.000)
Control variables	No	Yes	No	Yes
Province fixed effects	No	Yes	No	Yes
Year fixed effects	No	Yes	No	Yes
$\chi^{2}$	166.7	2114.9	162.3	1564.8
Prob> $\chi^{2}$	0.000	0.000	0.000	0.000
N	58,169			

Standard errors are in parentheses; * indicates significance levels * *p* < 0.1, ** *p* < 0.05, *** *p* < 0.01.

Model (7) shows that years of education are positively correlated with BMI, with a coefficient of 0.049, and significant at the 1% level. This indicates that an increase in years of education is positively correlated with having a BMI within the standard range. It suggests that individuals with higher levels of education are more likely to maintain standard body weight. After controlling for other variables (Model 8), this effect remains significant, and the coefficient slightly increases to 0.059. It indicates that the effect not only remains stable after controlling for other factors but also strengthens. This differs from the baseline regression results.

In Model (9), there is no significant relationship between years of education and the probability of having chronic diseases; however, after controlling for other variables (Model 10), the fitted value of years of education has a significant negative correlation with the probability of having chronic diseases, with a coefficient of −0.012. This demonstrates after controlling for endogeneity and other influencing factors an increase in years of education significantly reduces the probability of chronic diseases in individual.

Table 8 presents the second-stage results of the instrumental variable estimation of the impact of years of education on individual health relative deprivation levels. Model (1) is a simple model without control variables, Model (2) includes control variables, and Model (3) further controls for fixed effects of region and time. In Model 1 the estimated coefficient for years of education (fitted values) on health deprivation is −0.006 and significant at the 1% level. This indicates that without controlling for other variables, an increase in years of education leads to a reduction in the level of health deprivation. In Model 2 the coefficient for years of education is −0.004, also significant at the 1% level. It suggests that even when considering other influencing factors an increase in years of education remains associated with a reduction in health deprivation levels. Model 3 considers fixed effects for region and time, with the coefficient slightly decreasing to −0.003, yet still highly significant. This demonstrates that even after controlling for regional differences and time trends, the increase in years of education still significantly correlates with a reduction in health deprivation levels. The coefficient for gender in Models (2) and (3) shows significant values (−0.030 and − 0.031), indicating that compared to males, females have higher levels of health deprivation. The constant term represents the baseline level of individual health deprivation.

**Table 8:** IV-2SLS regression results of education on health inequality: second stage.

	Relative health deprivation level (Kakwani index)		
	OLS		
	(1)	(2)	(3)
Eduyear (fitted values)	−0.006*** (0.000)	−0.004*** (0.000)	−0.003*** (0.000)
Gender		−0.030*** (0.004)	−0.031*** (0.004)
Age		0.004*** (0.000)	0.003*** (0.000)
Age^2^		0.000* (0.000)	0.000 (0.000)
Control variables	No	Yes	Yes
Province fixed effects	No	No	Yes
Year fixed effects	No	No	Yes
Constant	0.216*** (0.002)	0.147*** (0.012)	0.506*** (0.161)
$\chi^{2}$	1635.26	6017.24	6487.33
Prob > $\chi^{2}$	0.000	0.000	0.000
*N*	58,169		

Standard errors are in parentheses; * indicates significance levels * *p* < 0.1, ** *p* < 0.05, *** *p* < 0.01.

Overall, Table 8 reflects three key pieces of information, first, despite the reducing trend, the negative impact of years of education on individual health deprivation levels remains significant across all three models. It signifies a potentially universal positive effect of education in reducing relative health disadvantages at the individual level. These findings support the view that education contributes to reducing health inequalities at the individual level. Secondly, the significant negative relationship between gender and individual health relative deprivation indicates that females are at a health disadvantage, which should be considered when formulating health policies to accommodate gender heterogeneity. Lastly, the decrease in coefficients from Model (2) to Model (3) suggests that the beneficial effects of education on health may vary by region and over time.

### 3.1 Multidimensional robustness tests

Table 9 shows seven models following two approaches transforming the dependent and the explanatory variables to analyze robustness tests. Models (1) to (3) involve transformations of the outcome variable, while Models (4) to (7) involve transformations of the explanatory variable. Specifically, in Model (1), self-rated health is transformed from an ordinal categorical variable to a binary variable, where individuals rating their health between 3 and 5 are defined as healthy (coded as 1), and those rating between 1 and 2 are defined as unhealthy (coded as 0). In Model (2), the dependent variables are the scores from the Center for Epidemiological Studies Depression Scale (CES-D) and the Kessler Psychological Distress Scale (K6), which are continuous variables. Models (4) to (7) use categorical variables for educational stages as explanatory variables, setting up five dummy variables for no education, elementary, junior high, high school, college, and above. The dependent variables include self-rated health, whether BMI is within the standard range, mental health status, and the presence of chronic diseases. All models control for other variables and fixed effects for time and region.

**Table 9:** The utility of education on health status: robustness test.

	SRH (binary)	CES-D score	K6 score	SRH	Mental health	Bmihealth	Chronic disease	Relative health deprivation level (Kakwani index)
	Probit	OLS	OLS	Ordered probit	Probit	Probit	Probit	OLS
	(1)	(2)	(3)	(4)	(5)	(6)	(7)	(8)
Eduyear	0.016*** (0.013)	−0.287*** (0.013)	−0.120*** (0.007)					
Edutype								
Primary				0.256*** (0.020)	0.077*** (0.006)	−0.009* (0.005)	−0.052*** (0.005)	−0.023*** (0.004)
Junior				0.384*** (0.020)	0.118*** (0.005)	−0.001 (0.005)	−0.071*** (0.005)	−0.041*** (0.004)
Senior				0.279*** (0.030)	0.135*** (0.007)	0.034*** (0.007)	−0.039*** (0.008)	−0.043*** (0.006)
High and above				0.563*** (0.057)	0.162*** (0.012)	0.087*** (0.013)	−0.096*** (0.013)	−0.034*** (0.011)
Constant		32.713*** (0.775)	13.343*** (3.591)					0.335** (0.152)
*N*	58,169							

Standard errors are in parentheses; * indicates significance levels * *p* < 0.1, ** *p* < 0.05, *** *p* < 0.01 (0.152).

Model (1) shows that with each additional year of education, the probability of being rated as healthy increases by 1.6%. It indicates that higher levels of education are associated with better self-rated health status. This result is significant at the 0.01 level. Models (2) and (3) indicate that as years of education increase, individual depression scores, whether measured by CESD or K6, significantly decrease. It demonstrates that higher levels of education are associated with better mental health conditions. Compared to those with no education, individuals at the elementary stage show significantly better outcomes in self-rated health (coefficient of 0.256), mental health (coefficient of 0.077), and the likelihood of having chronic diseases (coefficient of −0.052) but perform worse in terms of whether their BMI is within the standard range (coefficient of −0.009). Individuals at the junior high stage perform relatively better in terms of self-rated health (coefficient of 0.384), mental health (coefficient of 0.118), and the absence of chronic diseases (coefficient of −0.071) but show no significant differences in BMI and chronic diseases. High school-educated individuals perform significantly better across all health indicators, especially in whether their BMI is within the standard range, with a coefficient of 0.034, significant at the 0.01 level. It implies that those with a high school education are more effective in maintaining their weight. Individuals with a college education or higher show significantly better performance across all health indicators, especially in self-rated health and chronic diseases, with coefficients of 0.563. It reveals that higher education levels help to reduce the risk of chronic diseases and improve individual health maintenance and self-perceived health.

Overall, the educational human capital has a significant immediate efficacy on the health levels of rural residents. The results demonstrate that the models are robust in estimating the immediate utility of educational human capital on the health levels of rural residents.

Based on the regression results for the impact of educational types on individual health relative deprivation levels, Model (8) shows significant differences in the impact of different education levels on health deprivation after controlling for other variables, regional and temporal fixed effects. Specifically, the coefficient for elementary education is −0.023, for junior high it is −0.041, and for high school and above it is −0.043, all statistically significant at the 1% level. This indicates that all else being constant, compared to individuals with no elementary education, elementary education reduces the level of health deprivation by 2.343%. Individuals with junior high and higher education experience a further significant reduction in health deprivation levels compared to those with just elementary education, decreasing by 4.067 and 4.283%, respectively. These findings further support the positive role of education in reducing individual health-relative deprivation. Junior high and higher levels of education appear to have larger health benefits than elementary schooling. This may be due to higher stages of education providing more knowledge and skills, which likely include better health awareness and more effective health management. It specifies that the benefits of education are cumulative across different educational stages. Each higher level of education further reduces health deprivation levels, suggesting that long-term educational investments have a progressive effect on improving population health.

Table 10 reports the immediate effects of education on health relative deprivation levels constructed based on the Yitzhaki Index and the Podder Index. It shows in basic regression or two-stage regression methods controlling for endogeneity, the coefficients for the health inequality indicators are consistently negative. This displays that receiving more education can effectively reduce an individual’s level of health-relative deprivation, thereby significantly reducing the level of health inequality. These results indicate that the model remains robust in estimating the immediate utility of educational human capital on the health inequality of rural residents.

**Table 10:** The utility of education on health inequality: robustness test.

	Baseline Regression				IV-2SLS					
	Yitzhaki index		Podder index		Eduyear		Yitzhaki index		Podder index	
	(1)	(2)	(3)	(4)	(5)	(6)	(7)	(8)	(9)	(10)
Eduyear	−0.430*** (0.021)	−0.308*** (0.025)	−0.013*** (0.001)	−0.010*** (0.001)						
Average eduyear at the county level excluding the individual (IV)					0.533*** (0.010)	0.493*** (0.011)				
Eduyear (fitted values)							−0.536*** (0.105)	−0.070 (0.157)	−0.010*** (0.003)	−0.002 (0.005)
Age	0.204*** (0.011)	0.006*** (0.000)	−0.007*** (0.002)	0.235*** (0.011)	0.007*** (0.000)
Age^2^	0.001 (0.001)	0.000*** (0.000)	−0.001*** (0.000)	0.001 (0.001)	0.001** (0.000)
Control variables	No	Yes	No	Yes	No	Yes	No	Yes	No	Yes
Province fixed effects	No	Yes	No	Yes	No	Yes	No	Yes	No	Yes
Year fixed effects	No	Yes	No	Yes	No	Yes	No	Yes	No	Yes
Constant	15.028*** (0.160)	34.104*** (11.216)	0.316*** (0.005)	0.791* (0.449)	2.948*** (0.066)	2.438*** (0.118)	15.6904*** (0.635)	33.825*** (11.243)	0.302*** (0.021)	0.785* (0.450)
*N*					58,169

Standard errors are in parentheses; * indicates significance levels * *p* < 0.1, ** *p* < 0.05, *** *p* < 0.01.

As previously mentioned, the Kakwani Index for a given group is equivalent to the Gini coefficient for that group. Using this characteristic, this article calculates the Gini coefficients at the county level and regresses them on the county’s average years of education, with the findings shown in Table 11. It is observed that after controlling for other variables and regional and temporal effects, an increase in the average years of education at the county level still significantly reduces the local average level of health inequality. This illustrates that at both the individual and regional levels, the accumulation of educational human capital has a significant and favorable impact on lowering health disparities.

**Table 11:** Robustness test based on regional-level health inequality.

	Health Gini index (at the county level)	
	(1)	(2)
Average eduyear at the county level	−0.017*** (0.000)	−0.008*** (0.000)
Constant	0.270*** (0.002)	0.138*** (0.024)
Control variables	No	Yes
Province fixed effects	No	Yes
Year fixed effects	No	Yes
*N*	209	

Standard errors are in parentheses; * indicates significance levels * *p* < 0.1, ** *p* < 0.05, *** *p* < 0.01.

## 4 Discussion

Education and health are two crucial determinants of the welfare of rural residents. Clarifying the interaction between these factors is essential for formulating relevant public education and health policies. However, due to the issue of endogeneity, the relationship between education and health remains contentious and unresolved. To verify the causal positive impact of education on health, this study employs a two-stage least squares (2SLS) regression within a causal inference framework, using the county average years of schooling (excluding the individual in question) as an instrumental variable (IV) for individual educational attainment.

The baseline regression results indicate that, after controlling for other variables, as well as regional and time-fixed effects, individual years of schooling significantly positively influence subjective health levels and reduce the probability of chronic illness. Specifically, controlling for all predetermined variables, an additional year of education increases the likelihood of rural residents reporting “healthy” and “very healthy” by 0.2 and 0.4%, respectively, while reducing the probability of reporting “fairly healthy,” “average,” and “unhealthy” by 0.1, 0.2, and 0.4%. Psychological health improves by 0.9%, and the probability of chronic illness decreases by 0.3%, with health inequality dropping by 0.3%. The 2SLS regression results show that a one-year increase in the county average years of education increases individual years of schooling by 0.497 years, which is statistically significant at the 0.01 level and meets the IV validity test. This confirms that county average years of schooling is a valid instrument for individual educational attainment as per our study’s requirements. The second-stage regression results further demonstrate that, after addressing endogeneity, the immediate impact of education on improving individual health and reducing health inequality remains consistent with the baseline regression. This demonstrates that education can improve both self-rated and psychological health while also effectively reducing chronic illness prevalence and health inequalities. The findings suggest a causally positive influence of educational attainment on improving health levels and reducing individual-level health inequality, even after eliminating endogeneity interference. These results remain robust when the explanatory and dependent variables are replaced.

The gender heterogeneity test shows that education significantly enhances health levels and reduces health inequality for both men and women, whether measured by self-rated health, psychological health, BMI, or chronic disease incidence, as well as by the relative deprivation index of health. The positive effects are more pronounced for women compared to men. Therefore, improving the educational attainment of rural residents, particularly women, is a crucial strategy for promoting rural public health and improving overall health conditions in rural areas. The income heterogeneity test further reveals that education has a universal positive impact on self-rated health, psychological health, and the reduction of chronic disease incidence, with the most significant effects observed in low-income groups. Regarding health inequality, education’s impact is greatest among middle-and low-income groups, indicating that education effectively reduces health inequality within these populations, so its influence is relatively weaker among high-income groups. In summary, these findings underscore the need to consider both gender and income disparities when formulating health and education policies. On the one hand, while developing interventions for women and low-income groups, resource efficiency should be prioritized to maximize health benefits while using limited educational funding. On the other hand, interventions for men and high-income groups should focus on the fairness of educational resource allocation and ensuring effective resource conversion.

The findings of this study not only align with previous research (9, 15, 20, 38) but also further demonstrate the positive role of education in enhancing psychological health and reducing health-related deprivation. Zajacova and Lawrence (9) emphasize that higher levels of formal education are universally associated with better health outcomes and longer lifespans, with significant variations observed across different gender and racial groups. This aligns with the findings of the present study, which highlights the positive effects of education on the health of women and low-income populations, underscoring education’s critical role in mitigating health inequalities (9). Furthermore, Zajacova and Lawrence (9) discuss gender differences in the impact of education on health, noting that women exhibit greater sensitivity to educational attainment. This observation provides robust support for the analysis of gender heterogeneity in this study. Unlike previous research, this study not only emphasizes the multidimensional effects of education on health but also highlights the influence of education quality and social context on health outcomes, thereby expanding our understanding of the relationship between education and health. Compared with the study by Li and Liu (38), this research extends the two-stage least squares (2SLS) analysis to explore the multidimensional impacts of education on health, particularly psychological health and health inequality. Moreover, this study reveals a more substantial positive effect of education on women and low-income groups, addressing the limitations of Li and Liu (38) analysis regarding gender and income heterogeneity. While Clark and Royer (7) did not find a significant impact of education on health in the context of the United Kingdom, this study, based on data from rural China, confirms the positive effects of education on health. These differences may stem from varying socioeconomic contexts and research methodologies, highlighting the complexity of education’s impact on health across different countries and regions. The study by Fu (15), analyzed the effects of the expansion of Chinese higher education. The author found that higher education significantly improved residents’ health and reduced unhealthy behaviors. Similarly, this study employs the instrumental variable method but focuses on the health and health inequality of rural residents, particularly low-income groups, thereby expanding the broad impact of education on health. Guo (20) research mainly emphasizes the importance of educational development in poverty reduction and promoting prosperity. This study examines education’s impact on health and highlights the vital importance of education in improving the health of rural populations and eliminating health disparities. It indicates that beyond economic returns and equity, the impact of education on health is also an important research direction, particularly in enhancing the welfare of rural residents. In summary, this study systematically articulates the importance of education in improving the health of rural residents and reducing health inequality through comparisons and analyses with existing literature. Compared with prior research, this study not only broadens the understanding of education’s multidimensional impacts on health but also emphasizes the significant role of education in addressing gender and income heterogeneity. These findings provide more comprehensive empirical support for public policy formulation, suggesting that improving educational levels in rural areas is crucial for enhancing overall social health and reducing health inequality.

Theoretically, this study further validates the resource substitution theory (6), indicating that in resource-scarce rural areas, education, as a compensatory resource, significantly enhances health levels. Furthermore, the research confirms the causal effect of education in improving individual health and reducing health inequality. This finding provides new empirical support for current theoretical research on the relationship between education and health. Furthermore, it offers new insights into the specific mechanisms through which education affects health outcomes. Particularly in resource-constrained environments, the role of education is more prominent, providing crucial health support to disadvantaged groups and thereby reducing social health inequality. Practically, the findings of this study have significant implications for educational policy formulation. The results show that the positive impact of education on health is particularly pronounced among low-income and female groups. This suggests that, from an efficiency perspective, future education policies should prioritize resource allocation to these regions and groups to maximize health improvements. As China’s aging population problem intensifies, education, by improving health levels has the potential to alleviate pressure on the public healthcare system and positively impact the economic and social welfare system.

Future research should investigate the specific processes in which schooling improves health by changing lifestyle and behavior; specifically the interaction of many socioeconomic factors and education, such as how social support and the work environment affect wellbeing. Additionally, given regional disparities future research should delve into the distribution and utilization efficiency of health resources across different regions and optimize policies to reduce health inequality. Finally, future studies should focus on the long-term effects of education on specific health outcomes, such as the health status and quality of life of the older adult, to better understand the long-term effects of education on health.

## Data Availability

Since 1953, China has conducted a total of seven nationwide censuses, implemented by the National Bureau of Statistics. These censuses cover all 31 provinces, autonomous regions, and municipalities directly under the Central Government in mainland China. The statistical items have expanded from the initial population count, gender ratio, and age distribution to include education levels, occupational categories, family structures, housing conditions, migration and mobility, employment status, health conditions, fertility rates, and mortality rates. The most recent data is updated to 2020, which is the 7th national census. Data link: 7th National Census (2020) official website link: https://www.stats.gov.cn/zt_18555/zdtjgz/zgrkpc/dqcrkpc/. 6th National Census (2010) official website link: https://www.stats.gov.cn/zt_18555/zdtjgz/zgrkpc/d6crkpc/. For data from the other years, readers are welcome to request from the authors, and the raw data supporting the conclusions of this article will be made available by the authors, without undue reservation.
